# Cell cycle progression is an essential regulatory component of phospholipid metabolism and membrane homeostasis

**DOI:** 10.1098/rsob.150093

**Published:** 2015-09-02

**Authors:** Miguel Sanchez-Alvarez, Qifeng Zhang, Fabian Finger, Michael J. O. Wakelam, Chris Bakal

**Affiliations:** 1 Division of Cancer Biology, Chester Beatty Laboratories, Institute of Cancer Research, 237 Fulham Road, London SW3 6JB, UK; 2 Lipidomics Facility, Babraham Institute, Cambridge CB22 3AT, UK

**Keywords:** cell cycle progression, *Drosophila*, endoplasmic reticulum homeostasis, lipidomics, phospholipid metabolism, sterol response element binding proteins

## Abstract

We show that phospholipid anabolism does not occur uniformly during the metazoan cell cycle. Transition to S-phase is required for optimal mobilization of lipid precursors, synthesis of specific phospholipid species and endoplasmic reticulum (ER) homeostasis. Average changes observed in whole-cell phospholipid composition, and total ER lipid content, upon stimulation of cell growth can be explained by the cell cycle distribution of the population. TORC1 promotes phospholipid anabolism by slowing S/G2 progression. The cell cycle stage-specific nature of lipid biogenesis is dependent on p53. We propose that coupling lipid metabolism to cell cycle progression is a means by which cells have evolved to coordinate proliferation with cell and organelle growth.

## 1. Introduction

Cell growth and proliferation requires the *de novo* synthesis of plasma membrane and organelle endomembranes. The composition of the specific lipid species which make up cell and organelle membranes in both growing cells, and in daughter cells, is also of the utmost importance for cell homeostasis. For example, the relative amounts of key components such as phosphatidylethanolamine (PE) and phosphatidylcholine (PC) species are essential for the optimal function of the endoplasmic reticulum (ER) [1–4]. In addition, the levels of lipid subspecies with specific acyl chain variants profoundly affect biological phenomena as diverse as macrophage differentiation, early embryo development and fertility [5–9].

In all eukaryotes the Protein Kinase B-Target of Rapamycin (PKB/AKT–TOR) pathway promotes phospholipid anabolism by activating sterol response element binding proteins (SREBPs), which are key transcriptional controllers of lipid and phospholipid metabolism. The AKT–TOR pathway also promotes phospholipid anabolism by regulating lipolysis and autophagy [10–16]. We have recently demonstrated that TOR–SREBP regulation of lipid metabolism is required for ER homeostasis [17]. Thus, in response to growth factors such as insulin, AKT–TOR coordinately upregulates protein translation and lipid anabolism [11,16,17]. But it still remains largely unclear as to how activation of AKT–TOR–SREBP signalling is coordinated with cell cycle progression in order to promote membrane homeostasis during growth and division.

While clearly lipid anabolism must be integrated with increased translation and DNA synthesis during growth and cell cycle progression in order to ensure daughter cells have similar lipid content to mother cells, the act of cell division itself also involves profound changes in the architecture of cell membranes [18–21]. For example, cytokinesis is driven by changes in the levels of several lipid species, which have specific roles in the stepwise assembly and dynamics of regulatory complexes and cytoskeletal structures [22,23]. Consistent with a role of specific lipid species during cell proliferation, a number of early studies have suggested that the metabolism of specific lipids and phospholipids may be regulated in cell cycle specific fashions [20,21,24–26], and even demonstrated direct roles for cell cycle regulators such as the checkpoint factor Cdk1/Cdc28 in the control of lipid metabolism and trafficking in yeast [27]. But how lipid metabolism is regulated during periods of increased growth, such as during the G1 phase of the cell cycle, versus during other cell cycle phases, is very poorly understood.

Here, we show that lipid metabolism is tightly coordinated with cell cycle progression in metazoan cells. The production of key phospholipids that are essential for cell/organelle growth and homeostasis occurs during distinct phases of the cell cycle. Specifically, the G1/S transition is essential to sustain the balance of specific PC and PE species. Cells unable to progress through the G1/S transition are able to generate biomass *de novo*, but are unable to regulate PC and PE levels, which leads to ER stress. Such ER stress can be rescued through the exogenous supplementation of the relatively short, unsaturated fatty acid oleate (C18 : 1). We show that TOR-SREBP signalling is necessary, but not sufficient, for the regulation of lipid metabolism during growth and proliferation, as SREBP targets are fully activated only in cells that can progress through the G1/S transition. Furthermore, TOR promotes lipid anabolism not only by direct activation of SREBP, but also by regulating cell cycle progression itself. The compartmentalization of lipid metabolism across the cell cycle stages is dependent on p53 activity, because depletion of this regulator allows G1/S-stalled cells to synthesize the required phospholipid species and relieves ER stress derived from G1/S arrest. Moreover, analysis of isolated G1 versus S/G2 populations is compatible with a model by which lipid composition changes observed in insulin-treated cells are explained by changes in cell cycle distribution of the population. Kinetic control of cell cycle progression is thus an additional regulatory layer of lipid metabolism that is integrated with membrane homeostasis programmes.

## 2. Results

### 2.1. G1/S blockade during growth stimulation leads to defective lipidostasis and endoplasmic reticulum stress

**Figure 1.**
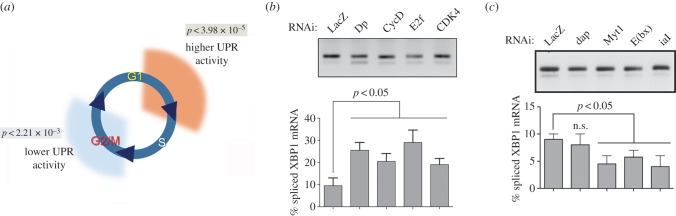
Cell cycle progression is integrated with ER homeostasis. (*a*) Genome-scale RNAi screens revealed a significant association of cell cycle progression with the control of ER homeostasis. Depletion of G1/S-positive regulators increased UPR signalling and depletion of G2/M progression regulators decreased basal ER stress. *P*-values denote enrichment significance for each functional class among hit lists [17]. (*b,c*) Regulators of G1/S progression (*b*) and G2/M progression (*c*) differentially impact ER homeostasis, as assessed by levels of IRE1-dependent splicing of XBP1 mRNA. Total RNA was isolated from S2R+ cells transfected for the indicated times with specific dsRNAs and semi-quantitative RT-PCR analysis was performed for XBP1 mRNA species (upper band, unspliced XBP1 mRNA; lower band, spliced XBP1 mRNA). Quantitation is derived from three independent experiments.

We recently performed genome-scale RNA interference (RNAi) screens in *Drosophila* cells for genes whose depletion increases, or decreases, activation of the Inositol Requiring Enzyme 1-X-box Binding Protein 1 (IRE1-XBP1) pathway, which is triggered upon induction of ER stress. We found that depletion of genes that promote G1/S transition upregulate the Unfolded Protein Response (UPR), depletion of genes that promote G2/M transition downregulate the UPR (figure 1*a*; also see [17]). We validated these observations by real-time polymerase chain reaction (RT-PCR) analysis of endogenous XBP1 splicing, key regulators of cell cycle progression. Depletion of G1/S-positive regulators, such as Cyclin D (CycD), Dp, E2f transcription factor or the cyclin-dependent kinase 4 (CDK4) all resulted in increased levels of IRE1-dependent splicing of XBP1 (figure 1*b*). Conversely, depletion of different proteins required for G2 progression and mitotic entry such as the IplI-aurora-like kinase/aurora kinase B (ial) and polo kinase were associated with lower levels of IRE1 activity as compared with mock-transfected cells (figure 1*c*). Secondary screens further suggested that cell cycle control integrates with lipid metabolism through the action of SREBP, to ensure ER homeostasis [17]. These observations supported a key role of cell cycle regulatory networks in the control of lipid metabolism and ER homeostasis.

**Figure 2.**
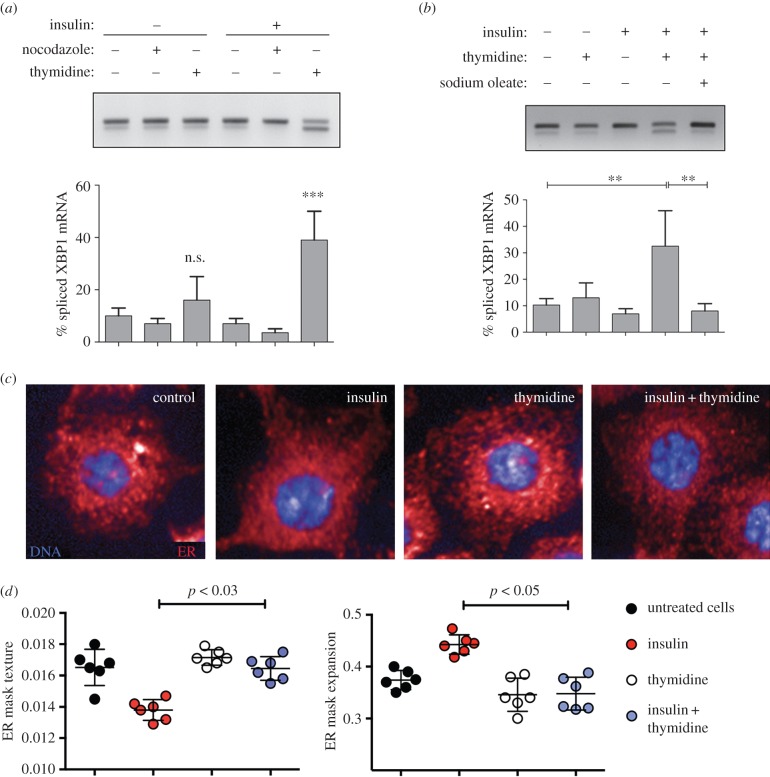
Growth signalling requires progression to S phase to ensure ER homeostasis and the remodelling of ER architecture. (*a*) Stimulation of growth signalling pathways by insulin is associated with loss of ER homeostasis upon blockade of G1/S progression. S2R+ cells (10^6^/ml density) were grown in the absence or in the presence of 500 nM insulin, and blocked from undergoing G1/S transition (2 mM thymidine) or G2/M transition (20 µM nocodazole). Total RNA was extracted and analysed by RT-PCR for IRE1-dependent XBP1 splicing (upper band, unspliced XBP1 mRNA; lower band, spliced XBP1 mRNA). (*b*) The induction of ER stress associated with the simultaneous stimulation of growth signalling and blockade of G1/S (500 nM insulin plus 2 mM thymidine, 18 h) can be rescued by the exogenous supplementation of unsaturated free fatty acid oleate (0.25 mM, 6 h before harvesting). Total RNA was extracted and analysed by RT-PCR for IRE1-dependent XBP1 splicing (upper band, unspliced XBP1 mRNA; lower band, spliced XBP1 mRNA). n.s., Not significant, *: *p* < 0.05; *p* < 0.02; *p* < 0.005. (*c,d*) Quantitative imaging of S2R+ cells, treated as indicated, fixed and immunostained for ER. Automated image analysis was performed as detailed elsewhere [17]. Upper panels (*c*) show representative snapshots of cells grown under the indicated conditions (blue, DNA; red, calreticulin/ER). Quantitation graphs (*d*) were derived from well-averaged values from six biological replicates, each containing approximately 1500 correctly segmented cells. Error bars depict ±s.d.

We hypothesized that cell cycle regulators are unlikely to play a role by directly modulating IRE1-XBP1 signalling, but rather that defects in cell cycle progression lead to imbalances in lipid composition that render cells unable to meet the requirements for sustained cell growth and proliferation. Consistent with this notion we found that insulin stimulation, which promotes cell and ER growth, leads to ER stress in *Drosophila* cells unable to progress through G1/S, but has little effect on ER stress in nocodazole cells arrested at G2/M (figure 2*a,b*; electronic supplementary material, figure S1*b*). Insulin-mediated ER stress in thymidine-arrested cells is due to a deficiency in lipid metabolism because exogenous supplementation of relatively short unsaturated fatty acid species (sodium oleate, C18 : 1) can decrease ER stress in these cells (figure 2*b*) [3,17,28]. Insulin stimulation also further exacerbates the ER stress that occurs following RNAi-mediated depletion of the G1/S progression regulator Dp (electronic supplementary material, figure S1*c*,*d*). Thus, we reasoned that insulin stimulation increases the demand for lipid precursors needed for cell/ER growth, and G1/S progression is required to satisfy this increase.

We previously showed that insulin stimulation results in significant peripheral expansion and remodelling of the ER, and that insulin-driven changes in ER architecture are dependent on TOR and SREBP activity [17] (figure 2*c,d*). Therefore, we aimed to determine whether similar defects in ER structure occurred in cells sustaining ER stress during G1/S blockade by quantitative image analysis of the ER in single cells [17]. Thymidine exposure alone did not provoke significant alterations in ER morphology (figure 2*c,d*). Importantly, insulin-dependent changes in ER architecture—namely, an increased peripheral occupation of ER and diminished texture features—were disrupted by blocking the progression of cells through the G1/S boundary with thymidine (figure 2*c,d*) [17]. These observations further support the idea that progression through G1/S is required for ER homeostasis and ER expansion/remodelling in proliferating cells, presumably through controlling the supply of lipid species to sustain membrane synthesis.

**Figure 3.**
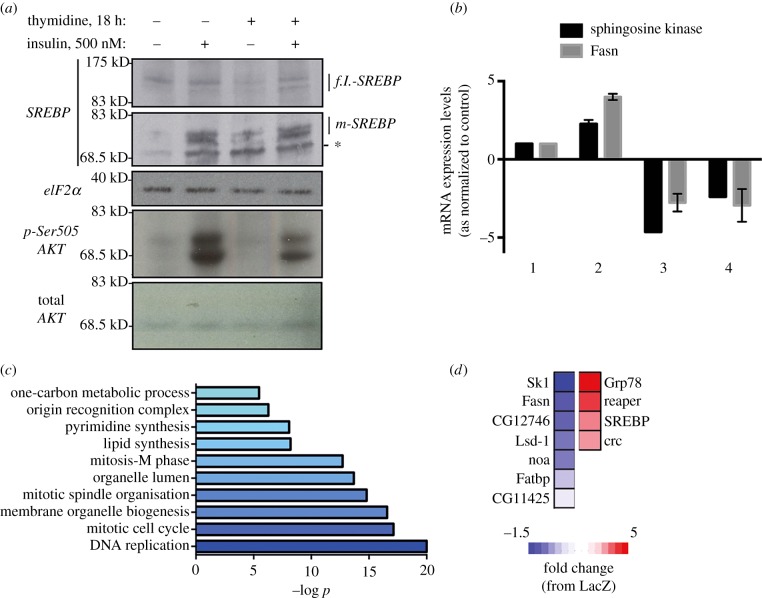
G1/S blockade neither provokes insulin resistance, nor attenuates SREBP processing, but diminishes SREBP-dependent transcriptional output. (*a*) Whole-cell lysates were obtained from cells grown under the indicated conditions, and analysed by western blotting for the indicated proteins. *: unspecific, low molecular weight band detected. (*b*) Total RNA was isolated from cells grown under the indicated conditions, and analysed by qRT-PCR for the expression levels of the indicated genes. 1: control S2R+ cells; 2: insulin (500 nM), 18 h; 3: thymidine (2 mM), 18 h; 4: insulin (500 nM)/thymidine (2 mM), 18 h. (*c*) Enrichment analysis of genes whose levels of mRNA are reduced (by at least 30%) in cells blocked at the G1/S boundary by RNAi-mediated depletion of Dp, as compared with wild-type cells. Total RNA was extracted and subjected to RNA array analysis from three independent samples per condition. (*d*) Fold change in mRNA expression in G1/S blocked cells for specific genes as compared with wild-type cells.

SREBP acts downstream of insulin-AKT–TOR signalling to regulate lipid metabolism, so that the availability of lipid building blocks such as specific fatty acid species, matches the demand for cell growth and concomitant ER expansion [11,16,17]. Thus, the fact that insulin stimulation increases ER stress in G1/S arrested cells, which can be prevented by addition of exogenous fatty acids, could be explained by dysfunctional SREBP activity during the G1/S blockade. To investigate SREBP activation and cell growth signalling during G1/S arrest, we compared, by western blot analysis, the pattern of relative activation of SREBP between normal and G1/S-blocked cells following insulin stimulation. Despite a moderate reduction (approx. 20%) as compared with unsynchronized cells, insulin stimulation still robustly activates AKT in G1/S-arrested cells, and results in cleavage of ER-localized SREBP to its nuclear localized form to levels equivalent to those of cells allowed to progress to G2/M (figure 3*a*). Paradoxically, qRT-PCR analysis revealed a dramatic decrease in mRNA levels of specific SREBP transcriptional targets, such as the sphingosine kinase 1 (Sk1) and the fatty acid synthase homologue (Fasn), in G1/S arrested cells, both in the absence or the presence of insulin stimulation (figure 3*b*). Microarray-based transcriptome profiling (figure 3*c*; electronic supplementary material, table S1) further revealed that, despite the cleavage and nuclear accumulation of SREBP in G1/S-arrested cells, targets of SREBP transcription were downregulated in cells unable to progress through G1/S (figure 3*d*). Thus, G1/S arrest does not directly affect insulin signalling or SREBP cleavage *per se*, but significantly affects the transcriptional output of cleaved SREBP. RNAi-mediated depletion of the master regulator of G1/S progression Dp resulted in a similar phenotype, demonstrating that G1/S progression is required for SREBP activity (electronic supplementary material, figure S2*a,b*). Taken together, our observations support a model whereby insulin signalling alone is necessary, but not sufficient (figures 1 and 2), for SREBP-dependent transcription in G1/S-arrested cells. Failure to activate SREBP in G1/S arrested cells dysregulates lipid metabolism, and leads to a subsequent loss of membrane homeostasis.

### 2.2. Insulin stimulation promotes a delay in S/G2-phase progression

**Figure 4.**
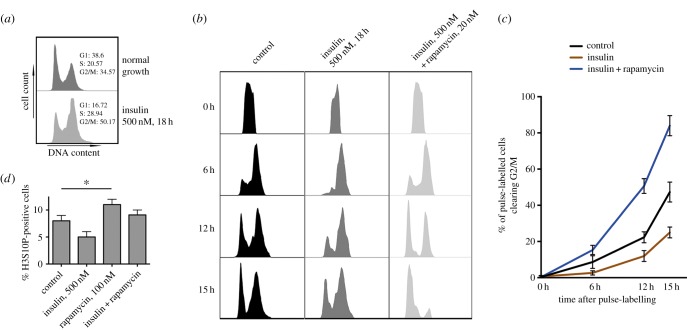
Insulin stimulation is associated with a delay in progression through S and G2 phases of the cell cycle. (*a*) Cell cycle profiles of S2R+ cells grown under either normal culture conditions (upper panel) or stimulated with 500 nM insulin for 16 h (lower panel). Estimated distributions across the cell cycle, according to Dean–Jett–Fox modelling, are shown. (*b*) Pulse-labelling with and immunostaining for bromodeoxyuridine allows for the profiling of progression through S/G2/M in cells grown under normal conditions (left column) or stimulated with 500 nM insulin approximately 4 h before starting the experiment. Estimated distributions across the cell cycle, according to Dean–Jett–Fox modelling, are shown. (*c*) The observed changes in cell cycle distribution of unsynchronized cultures is not derived from a delay or blockade in mitotic progression or cytokinesis as assessed by the mitotic marker phosphoserine 10-Histone H3. S2Rfl+ cells, grown in optical 384-well plates, were either left untreated or stimulated for 16 h with 500 nM insulin. pSer10HisH3 signal (normalized to cytoplasmic tubulin signal) was quantified and cells with an intensity greater than or equal to threefold the average background, denoting effective entry into mitosis, were counted from 12 replicate wells, each containing an total average approximately 2500 cells.

Because TORC1 signalling has been described as a key regulator of S/G2 progression kinetics [29], we hypothesized that the AKT–TOR pathway may regulate SREBP-dependent transcriptional output not only by directly promoting SREBP cleavage and nuclear translocation, but also indirectly by promoting cell cycle progression into a phase that is more permissive for SREBP activity. We profiled, using standard cell cytometry procedures, the cell cycle distribution of cells cultured in standard serum-containing growth media, as compared with cells exposed to growth media and insulin (figure 4*a*, upper panel). In the absence of insulin stimulation, approximately 40% of proliferating S2R+ cells are in G1, approximately 20.5% are in S-phase transition, and approximately 35% are in G2/M. Insulin stimulation leads to an approximate 25% increase in the number of S and G2/M cells, and a decrease in the number of G1 cells (approx. 20%) (figure 4*a*, lower panel). To determine how insulin stimulation affects cell cycle progression, we briefly pulse-labelled cell populations in S phase with the nucleotide analogue bromodeoxyuridine (BrdU) and estimated their progression time through subsequent stages of the cell cycle by flow cytometry (figure 4*b,c*) [29]. Untreated S2R+ cells spend approximately 10 h progressing from S through G2/M. However, insulin exposure significantly delayed the progression through these phases of the cell cycle by approximately 6 further hours (figure 4*c,d*). To determine the effects of insulin stimulation on G2/M progression, we stained normal and insulin-treated S2R+ cells for the early mitotic marker phosphoserine 10 of Histone H3 (figure 4*d*) [29], and observed that phospho-histone H3 levels were significantly *lower* in insulin-treated cells. Taken together these data demonstrate that insulin stimulation alters cell cycle progression by decreasing the rate of progression through S/G2 phase.

The TORC1-specific inhibitor rapamycin shortened the residency time of cells on S/G2 phase, and significantly reduced the delay induced by insulin exposure (figure 4*b–d*). Importantly, TORC1 inhibition significantly increased levels of phosphoserine 10 Histone H3 in a relatively short time period (12–18 h), further supporting the idea that TORC1 decelerates S/G2 progression (figure 4*b*, right bars).

### 2.3. Lipid mobilization in response to insulin is dependent on cell cycle stage

**Figure 5.**
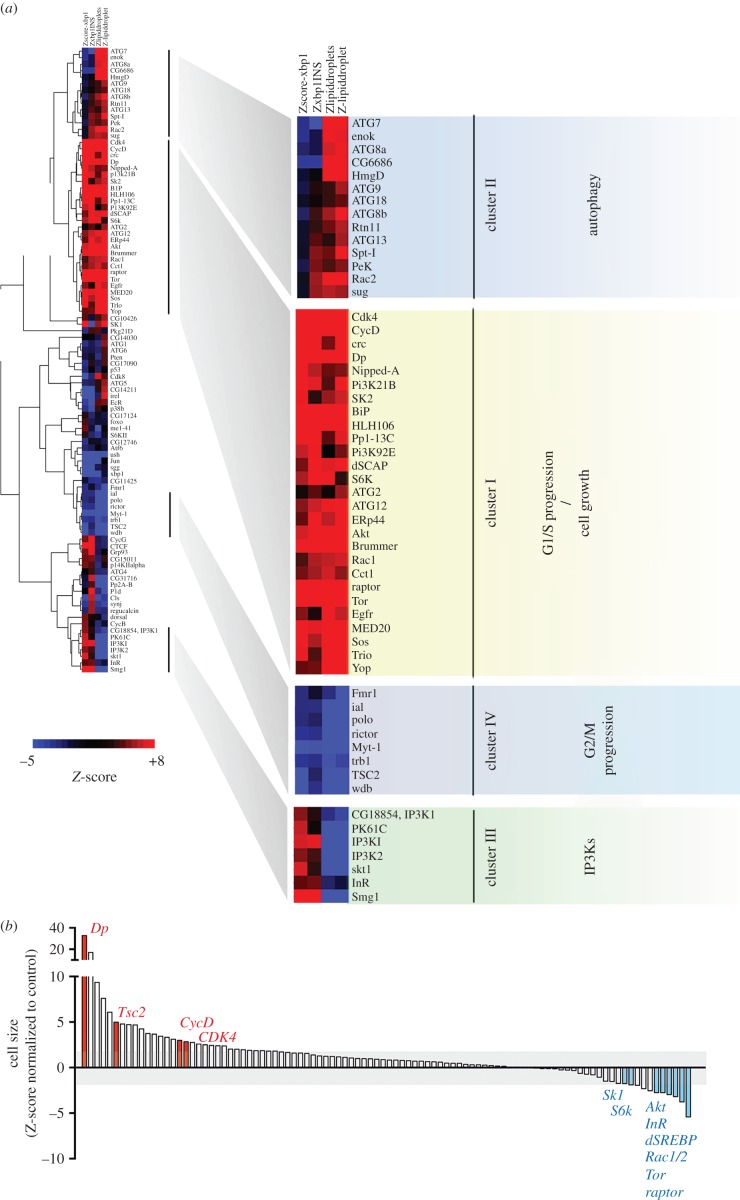
Blockade of progression through G1/S does not abolish cell growth, but is required for ER homeostasis and lipid mobilization in proliferating S2R+ cells. (*a*) Averaged *Z*-scores following depletion of each gene targeted by RNAi in the XH set sub-library were hierarchically clustered. Blown-up blocks (rightmost half of panel) show in higher detail selected clusters where the four *Z*-scores following gene depletion have a similarity value more than 0.78. The function of each cluster was manually annotated (coloured hue boxes). Columns 1–4, from left to right: 1: XBP1 splicing assay under normal growth conditions; 2: XBP1 splicing assay upon insulin stimulation (500 nM, 16 h); 3: lipid droplet relative accumulation of neutral lipids [17] under normal growth conditions; 4: lipid droplet relative accumulation of neutral lipids upon insulin stimulation. (*b*) Ranked cell size (normalized *Z*-scores) following the depletion of genes targeted by the XH sub-library [17]. Here nuclear size is a used a proxy for cell size. Some key genes controlling cell growth signalling, cell cycle progression and/or phospholipid metabolism are highlighted with red hue when their depletion increases average cell size, and with blue hue when depletion decreases it. The background grey box delimitates the significance cut-off of *Z* ± 2.

Our observations suggest that insulin regulates lipid metabolism by simultaneously activating SREBP and slowing cell cycle progression through S/G2 phases in a TORC1-dependent manner. In support of this idea, neutral lipids in proliferating S2R+ cells accumulate in droplets, but are mobilized in response to insulin stimulation [17]. However, we previously showed that insulin-mediated lipid mobilization does not occur in thymidine-treated cells, resulting in the accumulation of large lipid droplets [17]. We reasoned that if insulin-mediated lipid mobilization requires that cells progress through G1/S, RNAi manipulation of downstream components of the insulin signalling pathway and/or G1/S progression should also result in similar defects in lipid mobilization, and result in ER stress. Similarly, if lipid mobilization is enhanced in S/G2 phases, inhibition of G2/M-positive regulators should result in the deficient accumulation of neutral lipids. We thus tested how downregulation of 96 different genes, which we had previously identified as involved in the control of ER homeostasis and included several cell cycle regulators (‘XH set’, electronic supplementary material, table S2) [17], affected lipid mobilization and ER stress in the absence and presence of insulin. In parallel, we monitored ER stress across RNAi conditions ± insulin using the validated XBP1-EGFP reporter that recapitulates IRE1-dependent mRNA unconventional splicing [17]. In line with our hypothesis, depletion of canonical components of the insulin receptor (InR) signalling pathway such as TOR, AKT, Raptor or the Phosphatidylinositol-dependent kinase (PDK) homologue Pk61C, increased basal lipid accumulation (figure 5*a*), and greatly diminished mobilization in response to insulin. Importantly, depletion of the negative regulators of TORC1 signalling, such as TSC2 or PTEN, resulted in opposite phenotypes to depletion of TOR, AKT or PK61C, decreasing IRE1 steady-state activity and reducing accumulation of LDs (figure 5*a*).

Depletion of genes that promote G1/S progression such as CycD, CycE, cyclin-dependent kinase 4 (CDK4), deoxynucleotide kinase (dnk), and Dp phenocopied RNAi-mediated downregulation of positive growth factors, such as elevated IRE1 signalling and changes in the subcellular distribution of neutral lipids (figure 5*a*). Key positive regulators of G2 progression, which were identified in the screen as enhancers of ER stress, such as ial, Myt1 or polo, were also found as enhancers of lipid accumulation across both conditions. The fact that RNAi targeting most G1/S regulators leads to significant *increases* in cell size further supports our model that G1/S arrest *per se*, and not defects in insulin sensitivity or downstream signalling, leads to defects in lipid mobilization (figure 5*b*; see also figure 3*a,b*). Thus, while G1/S arrest does not block cell growth, this arrest probably results in the shunting of lipid precursors towards triacylglyceride synthesis and the formation of lipid droplets, and not, their incorporation to into phospholipids [27].

### 2.4. Phosphatidylcholine and phosphatidylethanolamine metabolism is regulated by cell cycle progression

**Figure 6.**
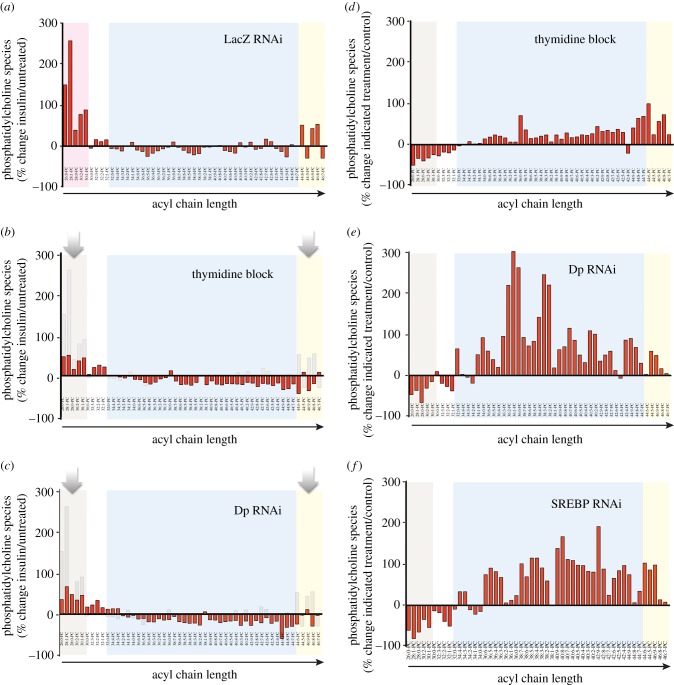
G1/S transition is required for insulin-mediated changes in phosphatidylcholine (PC) levels. (*a*) PC species of insulin-treated S2R+ cells as compared with normally cultured cells (expressed as a percentage). Three major groups of PC species are indicated with coloured backgrounds: magenta, short acyl chain species; cyan, medium-length acyl chain species; yellow, long acyl chain species (see main text). (*b*,*c*) Percentage of change in PC species upon insulin treatment in either thymidine arrested cells (*b*) or Dp depleted cells (*c*). The change in PC levels driven by insulin (*a*) is overlaid in light grey hue. Arrows highlights most changes occur in levels of short and very long acyl chain species (*d*,*e*) Change profile of PC species upon thymidine exposure (*d*) or Dp depletion (*e*) as compared to wild-type cells in the absence of insulin stimulation. (*f*) The differential profile observed following depletion of SREBP is shown for comparison [31]. Notably, depletion of SREBP in proliferation results in similar changes in overall PC levels as depletion of Dp in particular.

We next sought to investigate how lipid species are modified during insulin stimulation, and if the composition of lipid membranes is affected by G1/S arrest. We focused specifically on PC and PE species as (i) these are major constituents of plasma and ER membranes in eukaryotes [1,26]; (ii) as such, their relative levels in the ER membrane are major determinants of ER homeostasis [2,26,30]; and (iii) we [17] and others [31–33] have previously shown that their levels both regulate, and are regulated by, SREBP and the ER homeostatic machinery. Insulin stimulation of wild-type cells is associated with an increase in net cellular content of PC and PE species (electronic supplementary material, table S3). Upon species profiling, we also observed that insulin exposure leads to changes in the levels of particular PC and PE species. Specifically, insulin leads to an increase in shorter fatty acid chain PC species (12–18C), as well as a substantial relative decrease in a number of longer fatty acid chain species (20–24C), as compared with untreated cells (figure 6*a*; see also the electronic supplementary material, table S3). We interpret that, in normally proliferating cells, insulin positively regulates the *de novo* synthesis of specific, shorter fatty acid species that are directly incorporated into PE and PC pools.

Further supporting that G1/S-arrested *Drosophila* cells are not insulin-resistant, blockade of cell cycle progression at G1/S, either by thymidine or by RNAi-mediated depletion of the essential regulator Dp, did not abolish the increase in total PC levels upon insulin stimulation (electronic supplementary material, figure S3*a*; see also figure 3*a*). However, total PC was significantly diminished following insulin treatment, when cells were not allowed to progress through G1/S (electronic supplementary material, figure S3*b*). Furthermore, G1/S arrest also prevented the insulin-mediated changes in PC species composition that occur when cells are allowed to progress to S phase (figure 6*b,c*; electronic supplementary material, tables S4 and S5). For example, insulin stimulation in combination with G1/S arrest leads to a relative increase in a number of species of long fatty acid chains, including species bearing C24/C26 acyl chains (figure 6; electronic supplementary material, tables S4 and S5; right-hand arrows). We observed similar changes when profiling PE species from the same samples (electronic supplementary material, figures S3*a,b* and S4).

To determine whether G1/S arrest in itself results in dysregulation of PC and PE levels, we compared wild-type and G1/S arrested cells in the absence of insulin. Global levels of PC and PE species were significantly diminished across conditions that impaired G1/S progression in the absence of insulin stimulation (electronic supplementary material, figure S3*c*). In fact, the species that exhibited the largest increase upon insulin stimulation in wild-type cells (26 : 0/28 : 1/28 : 0) were also significantly depleted in unstimulated G1/S-blocked cells as compared with untreated wild-type cells (figure 6*d,e*). In fact, G1/S-blocked cells exhibit alterations in their profiles of PC and PE species that resemble those observed in SREBP-depleted cells (figure 6*d–f*; electronic supplementary material, table S6) [17]. We also observed very modest, but significant alterations in the overall degree of acyl chain saturation both in basal culture conditions as well as upon insulin exposure, when comparing wild-type cells with G1/S-arrested cells (electronic supplementary material, figure S3*d–f*). Altogether, these observations further support the idea that the G1/S transition is an integral component of the homeostatic response to insulin stimulation, and acts by regulating lipid metabolism.

### 2.5. Lipidomics profiling reveals cell cycle stage compartmentalization of lipid metabolism in normal proliferating cells

**Figure 7.**
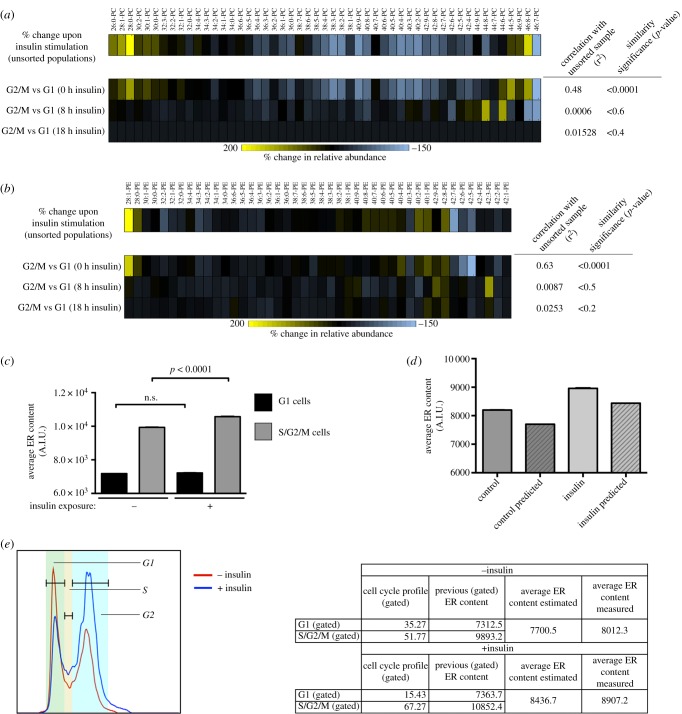
Population-level changes in phospholipid species following insulin stimulation can be partially explained by changes in cell cycle progression. (*a*,*b*) Heatmaps depict percent change in relative amounts of (*a*) PC species and (*b*) PE species. Top rows in (*a*) and (*b*) show changes resulting from insulin-stimulation in unsynchronized populations stimulated with insulin as compared with control cultures. Next three rows show changes in G2/M versus G1 cells following 0, 8 and 18 h of insulin stimulation. Similarities were assessed by pairwise correlation analysis and ANOVA analysis, across datasets derived from three biological replicates—values are indicated for each condition in the rightmost panels accompanying each graph. Changes in phospholipid levels between insulin stimulated cells and unstimulated cells are similar to the differences between G2/M and G1 (0 h insulin) cells, demonstrating that changes in cell cycle distribution (i.e. an enrichment of G2/M cells) can explain the differences in phospholipid levels following insulin treatment of asynchronous populations. Insulin stimulated cells become dissimilar from G2/M cells as they progress through the cell cycle (8 h and 18 h insulin). (*c*) ER content in G1 versus S/G2/M subpopulations, as gated by cell cycle profiling by Hoechst 33342 staining from control-treated or insulin-exposed cells. Data are derived from approximately 20 000 cells per condition; error bars report standard deviation. (*d*–*f*) ER content from control and insulin-treated unsynchronized populations of cells. (*d*) ER content as directly measured from ER tracker-counterstained cells (clear bars) or estimated from their cell cycle profiles and average contents from an independent measurement of gated G1 and S/G2/M populations. (*e*) A gating comparison for untreated and insulin-treated cells is shown. Data derived from three biological replicates.

We reasoned that if lipid metabolism is indeed a temporally restricted process, we should observe fluctuations in the levels of lipid species in normally proliferating cells in the absence of any chemically or genetically induced cell cycle arrest. Thus, we sorted live cells into G1 versus S/G2 cell cycle fractions by fluorescence-activated cell sorting (FACS) and analysed their relative composition in PC and PE species. We observed significant differences in the relative PC/PE composition of the membranes of G1 versus S/G2 cells. Notably, the difference in the lipid profiles between G1 cells versus S/G2 cells resembled the differences observed when comparing mixed populations of cells grown in normal conditions versus cells growing in the presence of insulin (figure 7*a,b*; see also figure 6*a–c*; electronic supplementary material, table S7). For example, S/G2-enriched fractions exhibit a higher proportion of short PC/PE species, and reduced levels of longer (24–26C) species, similar to insulin-treated, unsorted cultures (12–18C) (figure 7*a,b*). We further characterized the relative composition of PC and PE species in sorted G1 versus S/G2 subpopulations from cell cultures exposed for different times to insulin stimulation (figure 7*a,b*, blue and white bar sets). Of note, relative levels of PC/PE species in G1-sorted cells are indistinguishable from the relative composition of S/G2-sorted cells when derived from cell cultures stimulated for long periods of time with insulin. Because the composition of S/G2 cells did not change to a comparable extent across the three different insulin stimulation time points (electronic supplementary material, table S6), we suggest the residency time in specific stages of the cell cycle is an additional layer in the regulation of complex lipid metabolism.

Although the lipid composition we measure is derived from whole cells, we hypothesized that the architecture and composition of the ER membrane is also regulated by cell cycle progression. To discern how the cell cycle influences the ER membrane, we performed double-labelling experiments in live cells by simultaneously targeting DNA and ER contents with specific probes. As shown in figure 7*c*, S/G2 cells exhibit a higher average ER membrane content than G1 cells. Strikingly, insulin stimulation does not lead to comparable changes in the average ER content of each subpopulation (figure 7*c*). In fact, cell cycle distribution alone predicted the overall ER content of a non-segregated cell population (figure 7*d*,*f*; see figure 7*e* for the considered segmentation thresholds). These observations further suggest that lipid metabolism and ER homeostasis are regulated in a manner that is dependent on cell cycle progression.

### 2.6. P53 is required for the temporal asymmetry of phospholipid metabolism throughout the cell cycle

**Figure 8.**
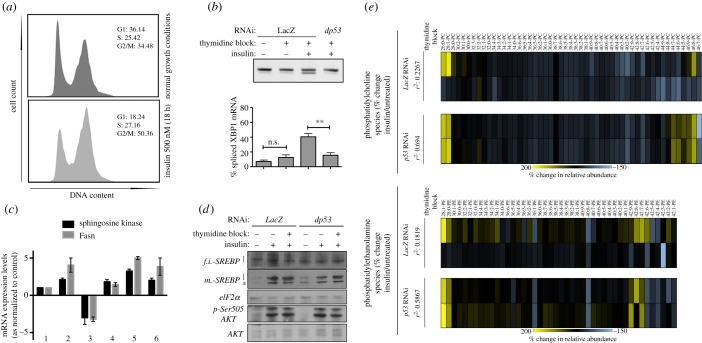
P53 suppresses cell cycle dependent SREBP activity. (*a*) Cell cycle profile of p53 depleted S2R+ cells in normal growth media (top panel), or following insulin stimulation (bottom panel). For comparison the cell cycle profile of normal S2R+ cells in the absence of proliferation is shown in figure 4*a*. (*b*) IRE1-dependent splicing of the XBP1 mRNA was estimated from RT-PCR analysis (upper band: unspliced XBP1 mRNA; lower band: spliced XBP1 mRNA) in insulin-treated G1/S arrested cells +/– p53 RNAi. (*c*) Normalized mRNA expression levels for the indicated genes in S2R+ subjected to the following treatments and RNAi transfections: 1: normal growth conditions/LacZ RNAi; 2: insulin (500 nM), 18h/LacZ RNAi; 3: insulin (500 nM)/thymidine (2 mM), 18h/Lac Z; 4: normal growth conditions/p53 RNAi; 5: insulin (500 nM), 18h/p53 RNAi; and 6: insulin (500 nM)/thymidine (2 mM), 18h/p53 RNAi. (*d*) p53 depletion does not affect growth signalling across the indicated conditions. S2R+ were transfected for 96 h with the indicated dsRNA preparations, treated as indicated, and lysed for western blot analysis. (*e*) Thymidine exposure abrogates phospholipid changes associated with insulin exposure in wild-type cells, but not in p53-depleted cells. Heatmap values represent percentage change in relative amounts of PC (upper block sets) and PE (lower block sets) species. For each phospholipid class, the upper heatmap block depicts data derived from mock-transfected cells and represents relative changes for each species upon insulin exposure, in the absence (upper row) or presence (lower row) of thymidine. The lower heatmap block of each phospholipid class depicts data derived from p53-depleted cells, and represents relative changes for each species upon insulin exposure, in the absence (upper row) or presence (lower row) of thymidine. Correlations for the phospholipid profiles between absence and presence of thymidine blockade are shown for each RNAi background and each phospholipid class.

We sought to identify factors responsible for partitioning of lipid metabolism into cell cycle stages. One candidate for such a factor is the transcriptional master regulator p53, whose activity is controlled in cell cycle dependent fashions and has been previously described to functionally interact in different contexts with SREBP [34–36]. In support of the notion that p53 may regulate lipid metabolism directly, and/or via control of cell cycle progression, we have previously observed that depletion of p53 results in ER stress, but only in cells simultaneously depleted of SREBP (electronic supplementary material, figure S5*a*,*b*) [17]. We decided to test whether p53 could be playing a role coupling cell cycle progression with lipid metabolism and ER homeostasis maintenance. Notably, p53 depletion by RNAi did not provoke significant changes in the cell cycle profile of normally cultured cells or insulin stimulated cells (figure 8*a*; see also figure 4). Thus, p53 does not function as a cell cycle gatekeeper during proliferation that occurs in the absence of any exogenous stress. However, p53 depletion consistently alleviated the ER stress caused by G1/S block in wild-type cells (figure 8*b*). Moreover, we observed p53 depletion allowed SREBP target genes to be expressed in G1/S-arrested cells to levels comparable to cycling cells (figure 8*c*). These effects are unlikely derived from differences in the regulation of the insulin-mediated signalling activity upstream of SREBP, because we did not observe significant changes when comparing wild-type cells with p53-depleted cells across different conditions in terms of AKT phosphorylation or SREBP cleavage (figure 8*d*).

We further profiled the PC and PE relative composition of cells stimulated to grow but blocked in G1/S, in the presence or the absence of p53 RNAi. p53 depletion completely rescued the alterations in growth-associated phospholipid metabolism associated with G1/S arrest, to the point that the levels of many phospholipid species recovered to levels comparable with those of insulin-stimulated cells that are competent to progress through G1/S (figure 8*e*; see also the electronic supplementary material, table S8). Taken together, our data suggest that p53 acts to attenuate SREBP-mediated lipid metabolism in G1/S arrested cells, but not during S/G2.

### 2.7. Endoplasmic reticulum stress results in a delay in progression through S/G2 phase

**Figure 9.**
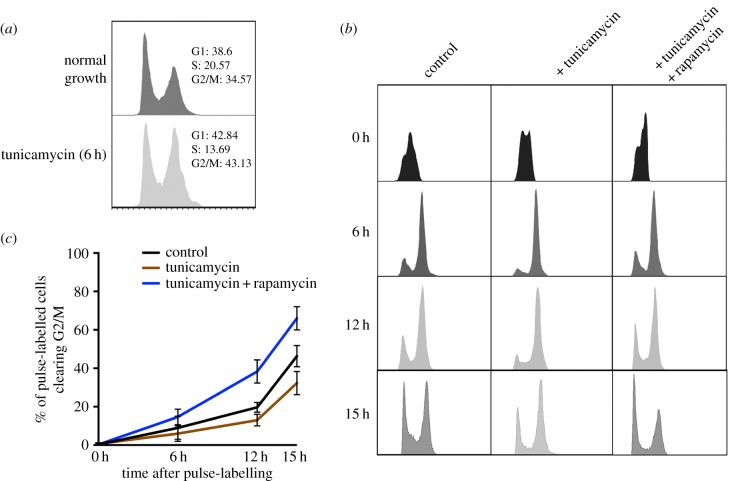
Acute induction of ER stress in S/G2 cells can induce a TORC1-dependent delay in mitotic progression. (*a*) Acute exposure of S2R+ cells to the ER stressor tunicamycin (Tm) leads to an accumulation of G2/M cells. (*b*) Cell cycle profiles of BrdU pulse-labelled control, Tm-treated and Tm + rapamycin cells. (*c*) Estimated cumulative percentage of BrdU-labelled cells clearing G2/M phases at the indicated time points. Data were derived from three biological replicates.

**Figure 10.**
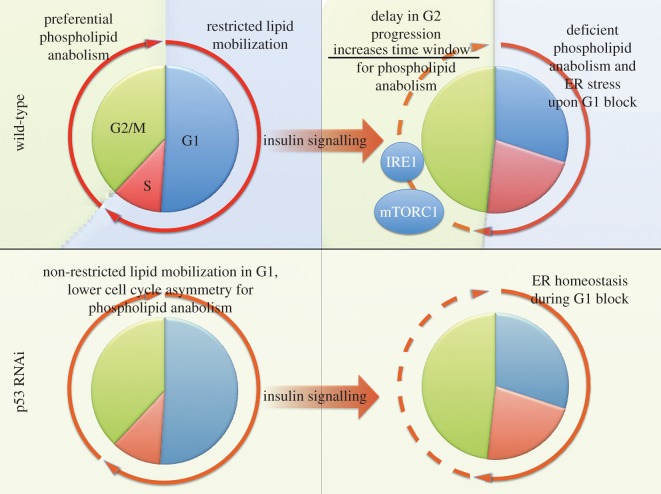
A model for p53-dependent integration of phospholipid metabolism and ER homeostasis maintenance with TORC1-dependent control of cell cycle progression.

Because TORC1-driven S/G2 progression kinetics appeared to be a component of the ER homeostatic response in *Drosophila* cells, we decided to test whether acute ER stress engages these mechanisms. We monitored S/G2 progression in the presence or the absence of the protein glycosylation inhibitor tunicamycin (Tm), which provokes acute ER stress in S2R+ cells in a time-scale of approximately 2 h (electronic supplementary material, figure S6). Acute exposure to Tm leads to an increase in G2/M populations (figure 9*a*). BrdU pulse-labelling experiments strongly suggested that these changes in cell cycle profile following Tm exposure are due to a delay in progression through S/G2/M phases (figure 9*b,c*). This cell cycle delay is likely TORC1-dependent because concomitant exposure to rapamycin largely abolished the cell cycle delaying effect of Tm (figure 9*b*, leftmost panel column; and figure 9*c*). We propose that challenges to ER homeostasis delay cell cycle progression in a TORC1-dependent manner in order to regulate lipid metabolism as part of a homeostatic response (figure 10).

## 3. Discussion

In this study, we show the regulation of PC and PE species, key building blocks of plasma and organelle membranes, are differentially regulated across the cell cycle. Furthermore, our observations support the hypothesis that the ‘residency time’ in specific stages of the cell cycle is an intrinsic mechanism that dictates the appropriate levels of specific lipid species. The underlying mechanisms involve SREBP dependent transcription, because we observe a pronounced attenuation in the transcriptional output of key SREBP targets upon G1/S arrest. SREBP is regulated both by AKT/TORC1-dependent signalling [11,12,14–16,31] as well as structural aspects of nuclear organization and cell cycle progression [15,37,38]. Here we show that both regulatory routes are essentially integrated, as full TORC1-mediated activation of SREBP requires TORC1-driven G2 entry and progression delay. Finally, we demonstrate that p53 is involved in coupling cell cycle progression to insulin-promoted lipid metabolism. A potential key component of this regulatory action could be an attenuation of SREBP transcriptional activity by p53, preferentially during G1/S stages of the cell cycle (figure 8*c*). The *Drosophila* homologue of p53 binds the promoter sequences of several SREBP targets such as *Fasn, Sk1* or *Cct1*, all of which are important regulators of lipid and phospholipid anabolism [39]. Moreover, p53 has previously been reported as a specific negative regulator of SREBP both *in vitro* and *in vivo* [36,40]. The physical and functional interplay between p53 proteins and SREBP is currently a major focus of attention in cancer metabolism biology [34,41]. To our knowledge, our observations suggest for the first time that cell cycle stage is a key contextual that decides the outcome of interactions between SREBP and p53.

Dysregulation in the balance of desaturated/saturated fatty acids (and their derivatives) in the ER membrane has been linked with alterations of ER homeostasis—presumably due to changes in the physical properties of the membrane itself [2,8,42–44]. The biological role of different lengths in the acyl chains, and their regulation, are less understood. Acyl chain length can have a profound impact on the curvature, fluidity and fusion rates of biological membranes [7]. Accumulation of longer acyl chain species has been previously linked with environmental or metabolic stress in yeast, and viral particle budding of hepatitis C [9]. This accumulation may promote higher stability of particular membrane structures [7,42,43]. The sharp increase in shorter PC and PE species upon acute insulin stimulation (figure 5*a*) may have an opposing effect, facilitating dynamic remodelling and expansion of the ER. A particularly intriguing possibility might be that specific acyl chain lengths are required for the mobilization of lipids in droplet stores [30,45,46]. In support of a model whereby specific acyl chain lengths are required for mobilization, impaired *de novo* synthesis of fatty acids, following depletion of SREBP or G1/S regulators, leads to simultaneous accumulation of neutral lipids in large, aberrant lipid droplets ([17,30,47] and this study).

Why may partitioning of lipid metabolism into distinct cell cycle phases be necessary for cell homeostasis? First, as one of the first steps in the Kennedy pathway—the main pathway for PC and PE synthesis in normally proliferating *Drosophila cells*—is the conjugation of choline to cytosine diphosphate [26,30], it is possible that partitioning of phospholipid metabolism throughout the cell cycle ensures phosphatidylnucleotide availability during or after S phase, where enhanced biosynthesis of nucleosides takes place [48,49]. Further supporting this idea, the AKT/TOR/SREBP axis itself is a major positive regulator of the pentose phosphate pathway and nucleoside metabolism, thus potentially integrating cell growth control, lipid/membrane biosynthesis, and nucleotide precursor metabolism [11,50]. Also, dysregulation of membrane synthesis and integrity maintenance can have a dramatic impact on the process of mitosis and cytokinesis [20,21,24]. Therefore, compartmentalization of lipid metabolism during cell cycle progression may act as a checkpoint strategy that ensures the changes in lipid metabolism required for cytokinesis occur only after successful duplication of genetic material [20,21,23,25,51].

Taken together we have shown there exists systems-coordination of cell growth, cell cycle progression, lipid metabolism and membrane homeostasis. Recent seminal studies suggest that SREBP is a node in metabolic networks where the actions of several oncogenes and tumour suppressors, such as effectors of the Wnt/Hippo pathway and p53, converge [34,41]. Together with the fact that loss of ER homeostasis has been recursively linked with cancer progression [52–54], this work could lead to a better understanding as to how aberrant metabolism and cell cycle dysregulation are integrated in cancer.

## 4. Material and methods

### 4.1. Cell culture and reagents

S2R+ cells were grown in Schneider's medium (Sigma) supplemented with 10% fetal bovine serum (FBS; Gibco) and 1× penicillin/streptomycin (Gibco) unless stated otherwise, at 25°C. Hoechst 33258 and 33342, bovine insulin, tunicamycin (Tm), thymidine, nocodazole, rapamycin and sodium oleate (Na-C18 : 1), and anti-bromouridine monoclonal antibodies were purchased from Sigma. Bodipy 493/510, ER Tracker Green, Alexa-488 and Alexa-647 immunoconjugates were purchased from Molecular Probes (Invitrogen). Phospho-histone H3 pSer10 and dAkt pSer505 antibody was purchased from Cell Signaling Technologies. RNAse A was purchased from Ambion. Antibodies against total *Drosophila* Akt and dSREBP have been developed by the Leevers and Rawson laboratories, respectively [3,17,29]. Calreticulin antibody and *α*-tubulin antibody were purchased from Abcam and SeroTec, respectively.

### 4.2. RT, qRT-PCR and protein analysis procedures, and RNA-microarray analysis

Detailed experimental methods have been described elsewhere [17]. The sequences of primers for qRT-PCR analysis can be found in [17]. Microarray analysis of gene expression described in figure 3*c,d* was performed from total RNA extracted in two consecutive steps of Trizol-chloroform purification. Total RNA was processed and assayed for gene expression in an Affymetrix 2.0 platform (Harvard Medical School) from three biological replicates (3 × 10^6^cells per replicate).

### 4.3. dsRNA synthesis, RNAi treatment and siRNA transfection

dsRNA synthesis was carried out using the MEGAScript T7 IVT kit (Ambion-Invitrogen) from T7 promoter-tailed PCR products, and purified using vacuum-driven 96-well filter plates (ThermoScientific). RNAi treatment through ‘bathing’ for RNAi screening was performed as described previously [55]. Batch transfection for dsRNA and DNA was performed using Effectene Reagent (Qiagen) following manufacturer's protocols. The following amplicons (www.flyrnai.org) were routinely used for validation and biochemical experiments: DRSC18359 (Raptor), DRSC37563 (SREBP), DRSC07402 (Dp), DRSC37618 (dp53), DRSC16655 (dE2f1), DRSC25031 (CycD), DRSC27263 (CDK4), DRSC07601 (dap), DRSC11228 (Myt), DRSC08509 (E(bx)) and DRSC03548 (ial).

### 4.4. High-throughput sample processing, automated image acquisition and analysis for functional genetic screenings

All automated sample processing and liquid handling was performed on a Cell::Explorer station (PerkinElmer and ThermoScientific). Methodologies and indicated specific procedures for dataset generation for figures 1, 2 and 4 (xbp1-EGFP splicing reporter, ER architecture analysis and lipid droplet assessment) have been published elsewhere [17]. Fixation and phospho-histone H3 pSer10 staining was performed as in previous reports [56]. Automated cell segmentation and image analysis were performed using the Acapella 2.5 analysis platform as follows: (i) segmentation of nuclei based on Hoechst 33258 signal; (ii) segmentation of cytoplasm region based on tubulin signal; (iii) filtering of artefacts based on low and high intensity thresholds, nuclear size and roundness, as well as removal of incomplete cell images; (iv) computation of intensities on each channel, normalization of pH3Ser10 signal to tubulin content; and (v) binning or counting of mitotic cells based on manually assessed thresholds.

### 4.5. Cytometry procedures

All analytic cytometry procedures were performed on an LSRII station (Becton Dickinson). Standard cell cycle profiling using propidium iodide (PI) staining was performed according to standard procedures. Briefly, approximately 2 × 10^6^ cells per condition were harvested by gentle scrapping and pipetting, washed once in cold Seecof saline buffer (SSB: 6 mM Na_2_HPO_4_, 3.67 mM KH_2_PO_4_, 106 mM NaCl, 26.8 mM KCl, 6.4 mM MgCl_2_, 2.25 mM CaCl_2_, pH 6.8), resuspended in 200 *μ*l of fresh SSB and completed to 1 ml and a final 80% ethanol, and stabilized overnight (O/N) at −20°C. After two washes in cold SSB, cells were finally resuspended in a freshly made staining mix (PBS1X, 0.1% Triton X-100, 40 µg ml^−1^ propidium iodide, 5 U ml^−1^ RNAse A). After segmentation of whole cells based on Forward Scatter and Side Scatter (FSC/SSC), PI signal intensity was normalized as DNA content by estimated size (depth) and frequency histograms were generated.

For pulse-labelling of cells entering S phase and immunostaining, we used a modified protocol from Wu *et al.* [29]. S2R+ cells treated with different conditions were exposed for approximately 10 min to 5 µM BrdU, and then gently washed with 2.5× volumes, two times in sterile SSB supplemented with 1% FBS and five times in fresh medium. Cells were then allowed to progress through cell cycle in the indicated conditions for the indicated times before processing for immunostaining. Cells were pelleted, washed twice in fresh SSB and ethanol-fixed as indicated for propidium staining. Nuclear pellets were then washed twice in fresh SSB, blocked and permeabilized in SSB-B (SSB, 0.5% TX-100, 2% BSA) for 1 h, and then incubated with a 1 : 20 dilution of anti-BrU antibody for 2 h at room temperature. After three washes in SSB-T (SSB, 0.1% TX-100) samples were incubated with 1 : 100 dilution of anti-mouse Alexa488 for 90 min, and then washed thrice in fresh SSB. Stain-positive cells were segmented and analysed for cell cycle progression.

Simultaneous labelling of nuclei and ER and ER-related structures was performed applying a modified protocol [57]. Briefly, live cells corresponding to each treatment were pulse labelled directly in the plate with 0.5 µg ml^−1^ Hoechst 33342 and 10 nM ER Tracker Green for 15 min. Cells were then gently scrapped and harvested and subjected to a brief step of trypsinization (0.5 U, 2 min), and then washed once in ice-cold complete medium. Cells were spinned and resuspended in ice-cold SSB supplemented with 0.25% BSA, and analysed for DNA and ER content signals.

Sorting of G1 versus S/G2 cells for lipidomics profiling (approx. 5 × 10^5^ sorted cells per condition and subpopulation) was performed on a cooled FACS Aria system based on Hoechst 33342 staining after harvesting, brief trypsinization and trypsin inactivation in fresh media on rotation for 30 min. Collection of sorted samples was on ice-cold SSB supplemented with 0.25% BSA before immediate pelleting and snap-freezing.

### 4.6. Lipid analysis

All data shown were generated from three independent biological replicates. Equal numbers of cells from each condition (approx. 10^6^ cells) were harvested by gentle pipetting, washed twice in fresh Seecof buffer and snap frozen in an ethanol/dry ice bath until further analysis. Upon thawing, cell pellets were subjected to Folch extraction and resuspended in 100 µl ethyl acetate/methanol 1 : 1. After appropriate dilution to work concentration, the lipid extract was analysed by positive ESI-MRM with an AB Sciex 4000 QTRAP station via loop injection with a Shimadzu Prominence HPLC autosampler. Pump A flow rate was set at 0.2 ml min^−1^ of mixed solvents chloroform/isopropanol/methanol/water 2 : 5 : 2 : 1 (volume ratio). Pump B flow rate was set at 0.05 ml min^−1^ of isopropanol. The mixed solvents A and B were used for PC and PE ESI ionization in a Turbo Spray ion source before MRM analysis. The operation parameters of the 4000 QTRAP for PC and PE analysis are detailed below.

PC analysis:
  — Source/gas parameters: curtain gas (CUR): 25; collision gas (CAD): medium; ion spray voltage (IS): 5500; temperature (TEM): 650; ion source gas 1 (GS1): 35; ion source gas 2 (GS2): 65; interface heater (ihe): on.
  — Compound parameters: declustering potential (DP): 140; entrance potential (EP): 10; collision energy (CE): 37; collision cell exit potential (CXP): 11. MRM time: 30 ms. Both Q1 and Q3 mass were set up at unit resolution.
PE analysis:
  — Source/gas parameters: curtain gas (CUR): 25; collision gas (CAD): medium; ion spray voltage (IS): 5500; temperature (TEM): 600; ion source gas 1 (GS1): 35; ion source gas 2 (GS2): 60; interface heater (ihe): on.
  — Compound parameters: declustering potential (DP): 90; Entrance Potential (EP): 10; collision energy (CE): 31; collision cell exit potential (CXP): 17. MRM time: 20 ms. Both Q1 and Q3 mass were set up at unit resolution.

For the quantitation of the relative PC/PE species shown across figures 6–8, we first calculated a weighted value for each species as compared with total amounts of PC or PE, respectively, and obtained averages of this normalized value across three independent biological replicates. Subsequently, a differential score was calculated for each species as the percentage change from the control average normalized value.

### 4.7. Data management, statistical analysis and analysis software

Data analysis, presentation and analysis of statistical significance were performed using the GraphPad Prism 6.0 software. For the quantification of XBP1 mRNA unconventional splicing by RT-PCR in the experiments shown in figures 1, 2 and 8, a minimum of two technical replicates, each including two biological replicates (hence, at least four independent runs), were analysed for Student's *t*-test for the indicated sample pairs. Cell cycle modelling (Dean–Jett–Fox algorithm) was performed using the FlowJo cytometry package using raw cytometry data. For the focused screens shown in figure 5, robust *Z*-scores were calculated for each gene using averaged values from replicates, and mean and standard deviation values from control cells. Hierarchical clustering of the dsRNA treatments according to their phenotypic signatures (figure 5*a*) was based on Euclidean distances.

## Supplementary Material

supplementary figures S1-5

## Supplementary Material

source data tables S1-8

## References

[1] Dobrosotskaya IY, Seegmiller AC, Brown MS, Goldstein JL, Rawson RB 2002 Regulation of SREBP processing and membrane lipid production by phospholipids in *Drosophila*. Science 296, 879–883. (doi:10.1126/science.1071124) 11988566 10.1126/science.1071124

[2] Fu S et al. 2011 Aberrant lipid metabolism disrupts calcium homeostasis causing liver endoplasmic reticulum stress in obesity. Nature 473, 528–531. (doi:10.1038/nature09968) 21532591 10.1038/nature09968 PMC3102791

[3] Seegmiller AC, Dobrosotskaya I, Goldstein JL, Ho YK, Brown MS, Rawson RB 2002 The SREBP pathway in Drosophila: regulation by palmitate, not sterols. Dev. Cell 2, 229–238. (doi:10.1016/S1534-5807(01)00119-8) 11832248 10.1016/s1534-5807(01)00119-8

[4] Walter P, Ron D 2011 The unfolded protein response: from stress pathway to homeostatic regulation. Science 334, 1081–1086. (doi:10.1126/science.1209038) 22116877 10.1126/science.1209038

[5] Ecker J, Liebisch G, Englmaier M, Grandl M, Robenek H, Schmitz G 2010 Induction of fatty acid synthesis is a key requirement for phagocytic differentiation of human monocytes. Proc. Natl Acad. Sci. USA 107, 7817–7822. (doi:10.1073/pnas.0912059107) 20385828 10.1073/pnas.0912059107 PMC2867858

[6] Kniazeva M, Shen H, Euler T, Wang C, Han M 2012 Regulation of maternal phospholipid composition and IP(3)-dependent embryonic membrane dynamics by a specific fatty acid metabolic event in *C. elegans*. Genes Dev. 26, 554–566. (doi:10.1101/gad.187054.112) 22426533 10.1101/gad.187054.112 PMC3315117

[7] Vrablik TL, Watts JL 2012 Emerging roles for specific fatty acids in developmental processes. Genes Dev. 26, 631–637. (doi:10.1101/gad.190777.112) 22474257 10.1101/gad.190777.112 PMC3323873

[8] Young RM et al. 2013 Dysregulated mTORC1 renders cells critically dependent on desaturated lipids for survival under tumor-like stress. Genes Dev. 27, 1115–1131. (doi:10.1101/gad.198630.112) 23699409 10.1101/gad.198630.112 PMC3672646

[9] Purdy JG, Shenk T, Rabinowitz JD 2015 Fatty Acid elongase 7 catalyzes lipidome remodeling essential for human cytomegalovirus replication. Cell Rep. 10, 1375–1385. (doi:10.1016/j.celrep.2015.02.003) 25732827 10.1016/j.celrep.2015.02.003 PMC4354725

[10] Aronova S, Wedaman K, Aronov PA, Fontes K, Ramos K, Hammock BD, Powers T 2008 Regulation of ceramide biosynthesis by TOR complex 2. Cell Metab. 7, 148–158. (doi:10.1016/j.cmet.2007.11.015) 18249174 10.1016/j.cmet.2007.11.015 PMC3882310

[11] Duvel K et al. 2010 Activation of a metabolic gene regulatory network downstream of mTOR complex 1. Mol. Cell 39, 171–183. (doi:10.1016/j.molcel.2010.06.022) 20670887 10.1016/j.molcel.2010.06.022 PMC2946786

[12] Laplante M, Sabatini DM 2010 mTORC1 activates SREBP-1c and uncouples lipogenesis from gluconeogenesis. Proc. Natl Acad. Sci. USA 107, 3281–3282. (doi:10.1073/pnas.1000323107) 20167806 10.1073/pnas.1000323107 PMC2840435

[13] Laplante M, Sabatini DM 2012 mTOR signaling in growth control and disease. Cell 149, 274–293. (doi:10.1016/j.cell.2012.03.017) 22500797 10.1016/j.cell.2012.03.017 PMC3331679

[14] Lewis CA, Griffiths B, Santos CR, Pende M, Schulze A 2011 Regulation of the SREBP transcription factors by mTORC1. Biochem. Soc. Trans. 39, 495–499. (doi:10.1042/BST0390495) 21428927 10.1042/BST0390495

[15] Peterson TR et al. 2011 mTOR complex 1 regulates lipin 1 localization to control the SREBP pathway. Cell 146, 408–420. (doi:10.1016/j.cell.2011.06.034) 21816276 10.1016/j.cell.2011.06.034 PMC3336367

[16] Porstmann T et al. 2008 SREBP activity is regulated by mTORC1 and contributes to Akt-dependent cell growth. Cell Metab. 8, 224–236. (doi:10.1016/j.cmet.2008.07.007) 18762023 10.1016/j.cmet.2008.07.007 PMC2593919

[17] Sanchez-Alvarez M, Finger F, Arias-Garcia del M, Bousgouni V, Pascual-Vargas P, Bakal C 2014 Signaling networks converge on TORC1-SREBP activity to promote endoplasmic reticulum homeostasis. PLoS ONE 9, e101164 (doi:10.1371/journal.pone.0101164) 25007267 10.1371/journal.pone.0101164 PMC4090155

[18] Lu L, Ladinsky MS, Kirchhausen T 2009 Cisternal organization of the endoplasmic reticulum during mitosis. Mol. Biol. Cell 20, 3471–3480. (doi:10.1091/mbc.E09-04-0327) 19494040 10.1091/mbc.E09-04-0327 PMC2719565

[19] Nakajima H, Yonemura S, Murata M, Nakamura N, Piwnica-Worms H, Nishida E 2008 Myt1 protein kinase is essential for Golgi and ER assembly during mitotic exit. J. Cell Biol. 181, 89–103. (doi:10.1083/jcb.200708176) 18378775 10.1083/jcb.200708176 PMC2287290

[20] Saitoh S et al. 1996 Aberrant mitosis in fission yeast mutants defective in fatty acid synthetase and acetyl CoA carboxylase. J. Cell Biol. 134, 949–961. (doi:10.1083/jcb.134.4.949) 8769419 10.1083/jcb.134.4.949 PMC2120970

[21] Witkin KL et al. 2012 The budding yeast nuclear envelope adjacent to the nucleolus serves as a membrane sink during mitotic delay. Curr. Biol. 22, 1128–1133. (doi:10.1016/j.cub.2012.04.022) 22658600 10.1016/j.cub.2012.04.022 PMC3381997

[22] Atilla-Gokcumen GE et al. 2014 Dividing cells regulate their lipid composition and localization. Cell 156, 428–439. (doi:10.1016/j.cell.2013.12.015) 24462247 10.1016/j.cell.2013.12.015 PMC3909459

[23] Echard A, Burgess D 2014 The changing lipidome during cell division. Cell 156, 394–395. (doi:10.1016/j.cell.2014.01.018) 24485447 10.1016/j.cell.2014.01.018

[24] Jackowski S 1994 Coordination of membrane phospholipid synthesis with the cell cycle. J. Biol. Chem. 269, 3858–3867. 8106431

[25] Jackowski S 1996 Cell cycle regulation of membrane phospholipid metabolism. J. Biol. Chem. 271, 20 219–20 222. (doi:10.1074/jbc.271.34.20219) 10.1074/jbc.271.34.20219 8702749

[26] Fagone P, Jackowski S 2012 Phosphatidylcholine and the CDP-choline cycle. Biochim. Biophys. Acta 1831, 523–532. (doi:10.1016/j.bbalip.2012.09.009) 23010477 10.1016/j.bbalip.2012.09.009 PMC3562404

[27] Kurat CF, Wolinski H, Petschnigg J, Kaluarachchi S, Andrews B, Natter K, Kohlwein SD 2009 Cdk1/Cdc28-dependent activation of the major triacylglycerol lipase Tgl4 in yeast links lipolysis to cell-cycle progression. Mol. Cell 33, 53–63. (doi:10.1016/j.molcel.2008.12.019) 19150427 10.1016/j.molcel.2008.12.019

[28] Kunte AS, Matthews KA, Rawson RB 2006 Fatty acid auxotrophy in *Drosophila* larvae lacking SREBP. Cell Metab. 3, 439–448. (doi:10.1016/j.cmet.2006.04.011) 16753579 10.1016/j.cmet.2006.04.011

[29] Wu MY, Cully M, Andersen D, Leevers SJ 2007 Insulin delays the progression of *Drosophila* cells through G2/M by activating the dTOR/dRaptor complex. EMBO J. 26, 371–379. (doi:10.1038/sj.emboj.7601487) 17183368 10.1038/sj.emboj.7601487 PMC1783464

[30] Krahmer N et al. 2011 Phosphatidylcholine synthesis for lipid droplet expansion is mediated by localized activation of CTP:phosphocholine cytidylyltransferase. Cell Metab. 14, 504–515. (doi:10.1016/j.cmet.2011.07.013) 21982710 10.1016/j.cmet.2011.07.013 PMC3735358

[31] Porstmann T et al. 2005 PKB/Akt induces transcription of enzymes involved in cholesterol and fatty acid biosynthesis via activation of SREBP. Oncogene 24, 6465–6481. (doi:10.1038/sj.onc.1208802) 16007182 10.1038/sj.onc.1208802

[32] Sriburi R, Bommiasamy H, Buldak GL, Robbins GR, Frank M, Jackowski S, Brewer JW 2007 Coordinate regulation of phospholipid biosynthesis and secretory pathway gene expression in XBP-1(S)-induced endoplasmic reticulum biogenesis. J. Biol. Chem. 282, 7024–7034. (doi:10.1074/jbc.M609490200) 17213183 10.1074/jbc.M609490200

[33] Sriburi R, Jackowski S, Mori K, Brewer JW 2004 XBP1: a link between the unfolded protein response, lipid biosynthesis, and biogenesis of the endoplasmic reticulum. J. Cell Biol. 167, 35–41. (doi:10.1083/jcb.200406136) 15466483 10.1083/jcb.200406136 PMC2172532

[34] Freed-Pastor WA et al. 2012 Mutant p53 disrupts mammary tissue architecture via the mevalonate pathway. Cell 148, 244–258. (doi:10.1016/j.cell.2011.12.017) 22265415 10.1016/j.cell.2011.12.017 PMC3511889

[35] Yahagi N et al. 2003 p53 Activation in adipocytes of obese mice. J. Biol. Chem. 278, 25 395–25 400. (doi:10.1074/jbc.M302364200) 10.1074/jbc.M302364200 12734185

[36] Yahagi N et al. 2004 p53 involvement in the pathogenesis of fatty liver disease. J. Biol. Chem. 279, 20 571–20 575. (doi:10.1074/jbc.M400884200) 10.1074/jbc.M400884200 14985341

[37] Bengoechea-Alonso MT, Ericsson J 2006 Cdk1/cyclin B-mediated phosphorylation stabilizes SREBP1 during mitosis. Cell Cycle 5, 1708–1718. (doi:10.4161/cc.5.15.3131) 16880739 10.4161/cc.5.15.3131

[38] Bengoechea-Alonso MT, Punga T, Ericsson J 2005 Hyperphosphorylation regulates the activity of SREBP1 during mitosis. Proc. Natl Acad. Sci. USA 102, 11 681–11 686. (doi:10.1073/pnas.0501494102) 10.1073/pnas.0501494102 PMC1187962 16081540

[39] Merlo P, Frost B, Peng S, Yang YJ, Park PJ, Feany M 2014 p53 prevents neurodegeneration by regulating synaptic genes. Proc. Natl Acad. Sci. USA 111, 18 055–18 060. (doi:10.1073/pnas.1419083111) 10.1073/pnas.1419083111 PMC4273405 25453105

[40] Bist A, Fielding CJ, Fielding PE 2000 p53 regulates caveolin gene transcription, cell cholesterol, and growth by a novel mechanism. Biochemistry 39, 1966–1972. (doi:10.1021/bi991721h) 10684646 10.1021/bi991721h

[41] Sorrentino G et al. 2014 Metabolic control of YAP and TAZ by the mevalonate pathway. Nat. Cell Biol. 16, 357–366. (doi:10.1038/ncb2936) 24658687 10.1038/ncb2936

[42] Volmer R, van der Ploeg K, Ron D 2013 Membrane lipid saturation activates endoplasmic reticulum unfolded protein response transducers through their transmembrane domains. Proc. Natl Acad. Sci. USA 110, 4628–4633. (doi:10.1073/pnas.1217611110) 23487760 10.1073/pnas.1217611110 PMC3606975

[43] Liu P et al. 2013 Membrane stress caused by octanoic acid in *Saccharomyces cerevisiae*. Appl. Microbiol. Biotechnol. 97, 3239–3251. (doi:10.1007/s00253-013-4773-5) 23435986 10.1007/s00253-013-4773-5

[44] Thibault G et al. 2012 The membrane stress response buffers lethal effects of lipid disequilibrium by reprogramming the protein homeostasis network. Mol. Cell 48, 16–27. (doi:10.1016/j.molcel.2012.08.016) 23000174 10.1016/j.molcel.2012.08.016 PMC3496426

[45] Walther TC, Farese RV Jr 2012 Lipid droplets and cellular lipid metabolism. Annu. Rev. Biochem. 81, 687–714. (doi:10.1146/annurev-biochem-061009-102430) 22524315 10.1146/annurev-biochem-061009-102430 PMC3767414

[46] Brasaemle DL, Wolins NE 2012 Packaging of fat: an evolving model of lipid droplet assembly and expansion. J. Biol. Chem. 287, 2273–2279. (doi:10.1074/jbc.R111.309088) 22090029 10.1074/jbc.R111.309088 PMC3268387

[47] Guo Y et al. 2008 Functional genomic screen reveals genes involved in lipid-droplet formation and utilization. Nature 453, 657–661. (doi:10.1038/nature06928) 18408709 10.1038/nature06928 PMC2734507

[48] Fridman A, Saha A, Chan A, Casteel DE, Pilz RB, Boss GR 2013 Cell cycle regulation of purine synthesis by phosphoribosyl pyrophosphate and inorganic phosphate. Biochem. J. 454, 91–99. (doi:10.1042/BJ20130153) 23734909 10.1042/BJ20130153

[49] Lane AN, Fan TW 2015 Regulation of mammalian nucleotide metabolism and biosynthesis. Nucleic Acids Res. 43, 2466–2485. (doi:10.1093/nar/gkv047) 25628363 10.1093/nar/gkv047 PMC4344498

[50] Robitaille AM et al. 2013 Quantitative phosphoproteomics reveal mTORC1 activates de novo pyrimidine synthesis. Science 339, 1320–1323. (doi:10.1126/science.1228771) 23429704 10.1126/science.1228771

[51] Babour A, Bicknell AA, Tourtellotte J, Niwa M 2010 A surveillance pathway monitors the fitness of the endoplasmic reticulum to control its inheritance. Cell 142, 256–269. (doi:10.1016/j.cell.2010.06.006) 20619447 10.1016/j.cell.2010.06.006 PMC3359143

[52] Bi M et al. 2005 ER stress-regulated translation increases tolerance to extreme hypoxia and promotes tumor growth. EMBO J. 24, 3470–3481. (doi:10.1038/sj.emboj.7600777) 16148948 10.1038/sj.emboj.7600777 PMC1276162

[53] Wang S, Kaufman RJ 2012 The impact of the unfolded protein response on human disease. J. Cell Biol. 197, 857–867. (doi:10.1083/jcb.201110131) 22733998 10.1083/jcb.201110131 PMC3384412

[54] Nakagawa H et al. 2014 ER stress cooperates with hypernutrition to trigger TNF-dependent spontaneous HCC development. Cancer Cell 26, 331–343. (doi:10.1016/j.ccr.2014.07.001) 25132496 10.1016/j.ccr.2014.07.001 PMC4165611

[55] Ramadan N, Flockhart I, Booker M, Perrimon N, Mathey-Prevot B 2007 Design and implementation of high-throughput RNAi screens in cultured *Drosophila* cells. Nat. Protocols 2, 2245–2264. (doi:10.1038/nprot.2007.250) 17853882 10.1038/nprot.2007.250

[56] Yin Z et al. 2013 A screen for morphological complexity identifies regulators of switch-like transitions between discrete cell shapes. Nat. Cell Biol. 15, 860–871. (doi:10.1038/ncb2764) 23748611 10.1038/ncb2764 PMC3712499

[57] Liang L, Haug JS, Seidel CW, Gibson MC 2014 Functional genomic analysis of the periodic transcriptome in the developing Drosophila wing. Dev. Cell 29, 112–127. (doi:10.1016/j.devcel.2014.02.018) 24684830 10.1016/j.devcel.2014.02.018

