# Supplementary material for: Cell cycle progression is an essential regulatory component of phospholipid metabolism and membrane homeostasis
Source: Open Biol. 2015 Sep 2;5(9):150093. doi: 10.1098/rsob.150093 (PMC4593667; doi:10.1098/rsob.150093)
Supplement: supplementary figures S1-5 [file rsob150093supp1.pdf]

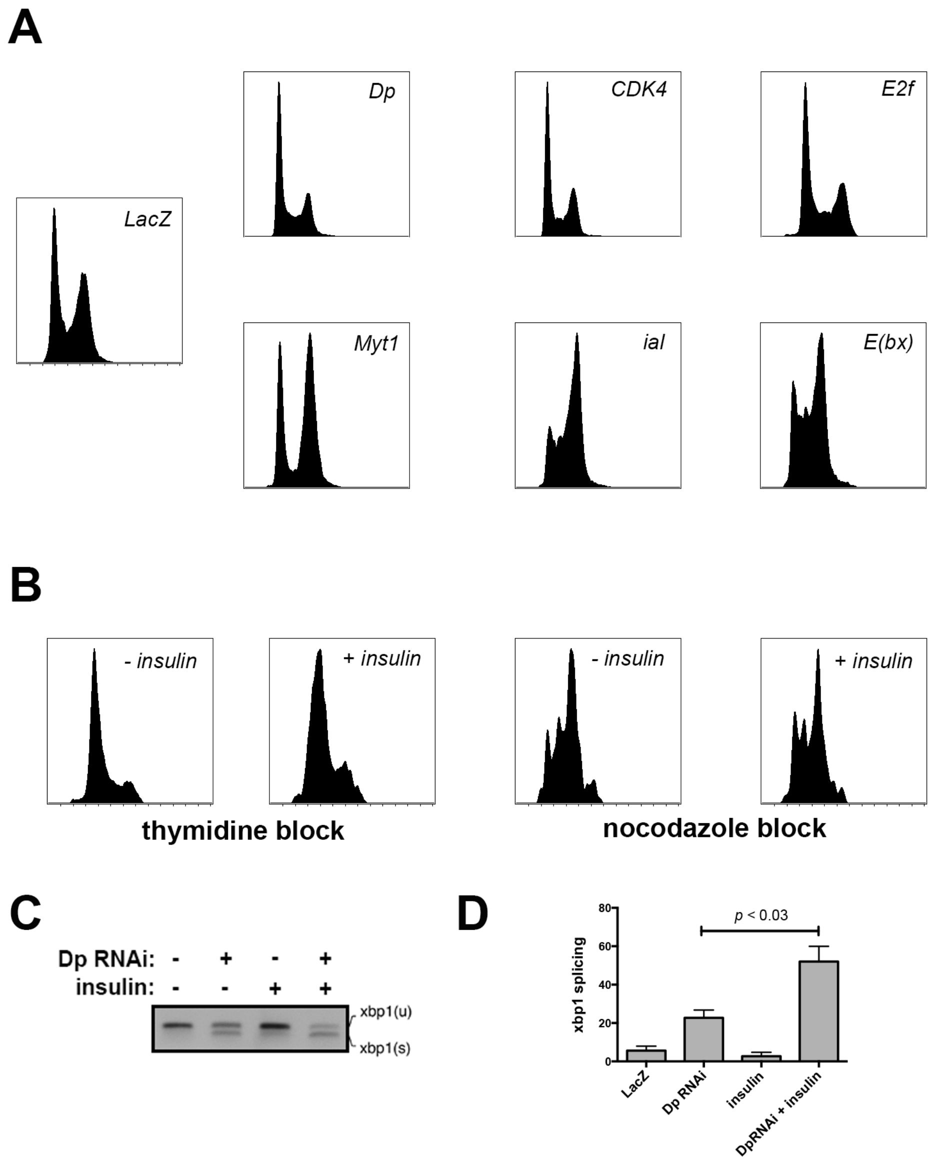

Figure S1- Sanchez-Alvarez et al.

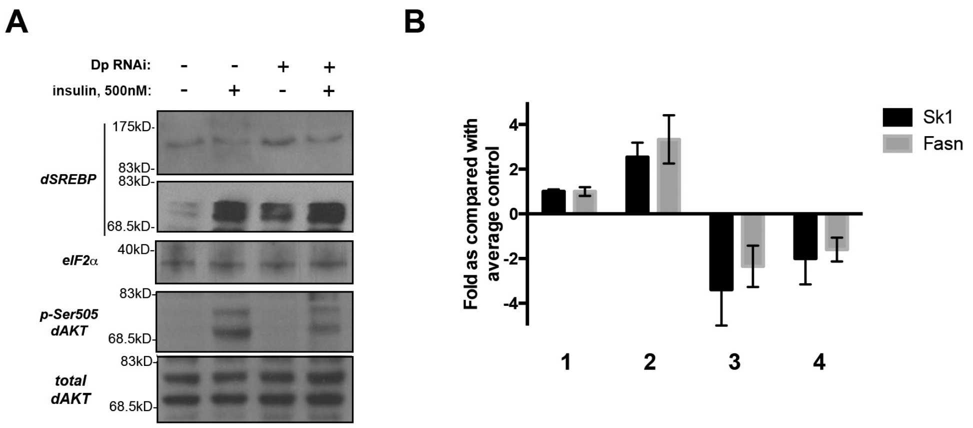

Figure S2- Sanchez-Alvarez et al.

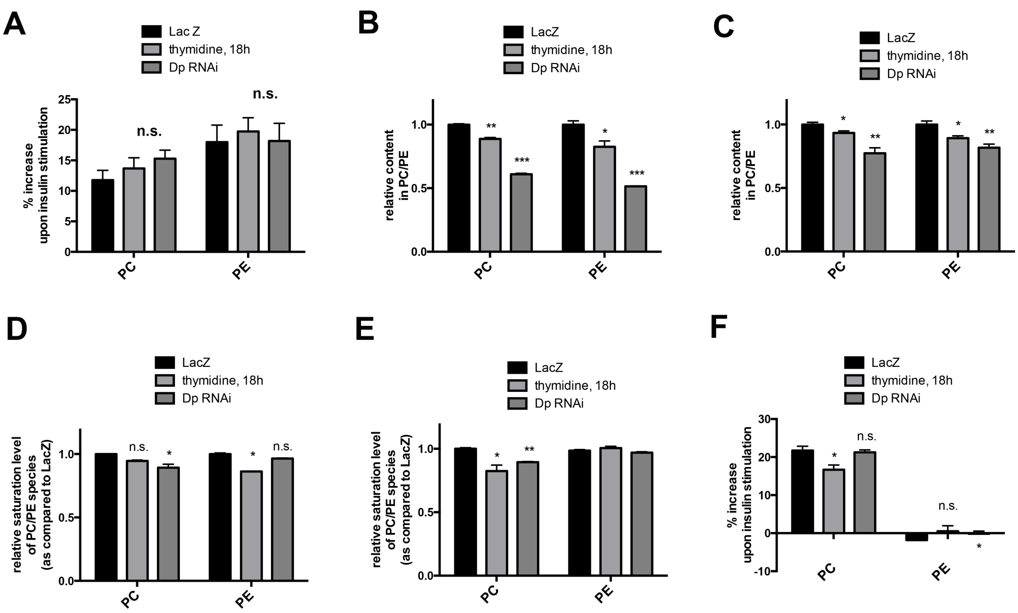

Figure S3- Sanchez-Alvarez et al.

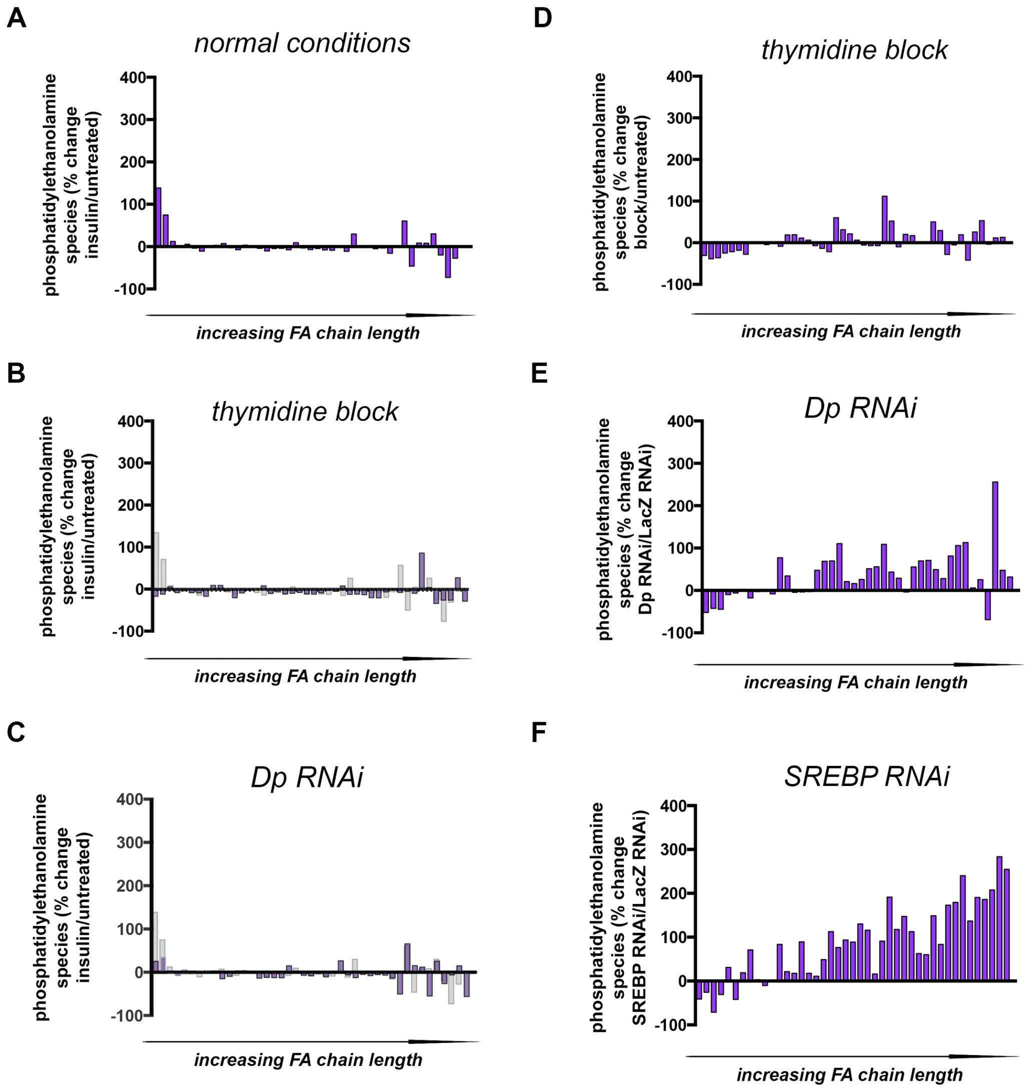

Figure S4- Sanchez-Alvarez et al.

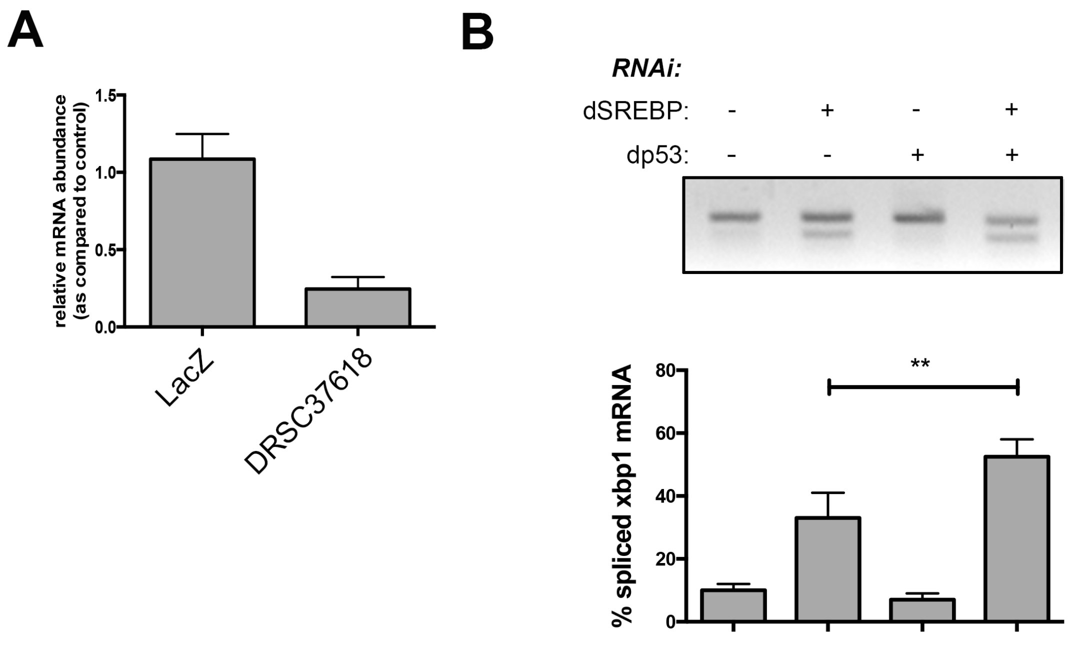

Figure S5- Sanchez-Alvarez et al.
